# Evaluating the Psychometric Properties of the Indonesian Version of the Stroke Stigma Scale Among Indonesian Stroke Survivors

**DOI:** 10.3390/ijerph23070918

**Published:** 2026-07-17

**Authors:** I Gede Juanamasta, Rapin Polsook, Bootan Ahmed, Yupin Aungsuroch, Ni Made Ratih Comala Dewi, I Gede Griya Suparta, Ferry Efendi

**Affiliations:** 1 Faculty of Nursing, Chulalongkorn University, Bangkok 10330, Thailand; 2 Frances Payne Bolton School of Nursing, Case Western Reserve University, Cleveland, OH 44106, USA; bootan.ahmed@case.edu; 3 Rumah Sakit Umum Daerah Bali Mandara, Denpasar 80227, Indonesiagsuparta5@gmail.com (I.G.G.S.); 4 Faculty of Nursing, Universitas Airlangga, Surabaya 60115, Indonesia

**Keywords:** stigma, stroke, scale, survivors, translation, Indonesia, reliability, validity

## Abstract

**Highlights:**

**Public health relevance—How does this work relate to a public health issue?**

- Stroke survivors may experience stigma as well as physical disability.
- This study examines a tool to measure stigma in Indonesian stroke survivors.

**Public health significance—Why is this work of significance to public health?**

- The study shows that the Indonesian Stroke Stigma Scale is valid and reliable.
- This tool can help identify stigma that may affect recovery and quality of life.

**Public health implications—What are the key implications or messages for practitioners, policy makers and/or researchers in public health?**

- Health workers can use this scale to detect stigma during stroke care and rehabilitation.
- The findings support stigma-sensitive care and future research in Indonesia.

**Abstract:**

Purpose: To assess the validity and reliability of the Indonesian Stroke Stigma Scale (SSS-I), including its four dimensions and 16 items, among Indonesian stroke survivors. Validation of the Stroke Stigma Scale (SSS) is recommended across various mental health contexts and community settings. However, construct validity, convergent validity, and reliability in particular have not been examined and validated among Indonesian stroke survivors. Materials and Methods: A total of 112 stroke survivors participated. The SSS was translated into Indonesian using Brislin’s back-translation method. Confirmatory factor analysis (CFA) was conducted to assess construct validity. Results: CFA indicated a good model fit, with factor loadings for all items exceeding 0.30. The convergent validity, represented by the average variance extracted, ranged from 0.507 to 0.666, suggesting that the latent constructs explained more than 50% of the variance. The scale demonstrated excellent reliability, with a total Cronbach’s alpha of 0.917 and composite reliability ranging between 0.752 and 0.887. Conclusions: The Indonesian version of the SSS demonstrated preliminary evidence of internal consistency and construct validity, supporting its use for assessing stigma among stroke survivors, with further validation recommended. This instrument helps healthcare providers to understand Indonesian stroke survivors’ needs, supports decision-making, and enhances outcomes.

## 1. Introduction

Stroke is the second leading cause of death (approximately 7 million deaths annually) and the third leading cause of combined death and disability (as expressed by disability-adjusted life-years lost [DALYs]; over 160 million DALYs) in the world [1]. According to the World Stroke Organization [1], the global cost of stroke is over USD 890 billion (0.66% of the global GDP). Between 1990 and 2021, the global burden of stroke increased significantly in terms of the total number of cases, with incident strokes increasing by 70%, stroke-related deaths by 44%, prevalent stroke cases by 86%, and disability-adjusted life years (DALYs) by 32%. Notably, the majority of this burden—87% of deaths and 89% of DALYs—was concentrated in low- and lower-middle-income countries [1].

In 2021, there were an estimated 11.9 million new strokes globally, with a total prevalence of 93.8 million stroke survivors, reflecting a 70% increase in incident strokes since 1990 [1]. Stroke survivors often face not only physical and cognitive challenges, but also significant psychosocial burdens, including stigma. Stigma is an “attribute that is deeply discrediting, and that reduces the bearer from a whole and usual person to a tainted, discounted, and inferior” [2,3]. Stigma is categorized into “public stigma” and “self-stigma,” both of which adversely impact patients’ self-esteem [4]. Misunderstandings held by family members, friends, and healthcare professionals negatively affect patients’ cognitive, emotional, and social functioning, potentially exacerbating depressive symptoms [4].

According to two studies in Indonesia, 63% of stroke survivors experience mild stigma and 37% experience moderate stigma according to the Stroke Stigma Scale (SSS) [5,6]. The SSS has also been translated into Japanese [7]. However, the construct validity of the SSS has not been validated in Indonesian stroke survivors.

Although the SSS has demonstrated acceptable psychometric properties in previous studies, its applicability across diverse cultural contexts remains underexplored. Cultural beliefs, social norms, and health-related stigma vary significantly between populations and can influence how individuals perceive and internalize stigma after a stroke. Indonesia, with its unique sociocultural, linguistic, and healthcare dynamics, represents a markedly different context from the populations in which the scale has been previously validated. Therefore, it is essential to reexamine the psychometric properties of the SSS among Indonesian stroke survivors to ensure its cultural relevance, conceptual clarity, and measurement validity.

Therefore, this study addresses this critical gap by evaluating the scale’s reliability, factor structure, and construct validity in an Indonesian context, thereby contributing to more accurate stigma assessment and potentially informing culturally tailored interventions for stroke survivors in Indonesia.

Stroke survivors are known to experience a wide range of health complications, from relatively mild issues to potentially lethal ones. For example, stroke survivors may continue to experience impairments to their functional capacities and quality of life up to three months following the event [8]. For up to a year following a minor stroke, survivors may experience some level of disability and need to make adjustments to their lifestyle [5,9]. There is a possibility that some stroke survivors may face significant and long-lasting cognitive impairment [10,11,12].

Pain, fever, infections, falls, sadness, anxiety, emotionalism, disorientation, exhaustion, etc., are only a few of the many post-stroke health issues that have been highlighted in previous research [13]. Stroke-related stigma is an issue faced by many stroke survivors, yet it has not received substantial attention. Stigma typically emerges when labeling, stereotyping, separation, loss of status, and discrimination occur simultaneously within a context of power imbalance [2,3].

One of the tools that has been developed to measure stigma among people living with stroke is the Stroke Stigma Scale (SSS) [14].

### 1.1. Review of the Literature

A study highlights the many challenges that stroke survivors face after the event [5]. These include physical disabilities like paralysis and incapacity to move and problems with speech, hearing, and vision. Survivors experience stress, anxiety, and shame over their condition, diminished family role, low self-esteem, depression, and pessimism about their chances of a full recovery. Stroke survivors who struggle to emotionally adjust to their symptoms are less likely to participate in rehabilitation programs and more likely to miss appointments with their doctors [15].

Stroke care and rehabilitation can be expensive, which can put a strain on families’ budgets and make them reluctant to seek medical attention when needed. The internal stigma that stroke survivors experience is a result of their condition and the negative thoughts of their loved ones about stroke, which leads to this situation [16]. According to Zhu et al. [17], there are two types of internal stigma: enacted stigma, which stems from real-life experiences of discrimination in social settings, and felt stigma, which is associated with feelings of shame, difference, or discrimination [17]. Ninety percent of stroke survivors in a Chinese study reported experiencing stigma and seventy-two percent reported acting on that stigma [18]. On the other hand, few studies have examined the effects of internal stigma on stroke survivors in Indonesia, even though a substantial proportion of Indonesian stroke survivors experience post-stroke stigma [5,6].

Stroke survivors may experience difficulty believing in their own abilities to recover and may lose motivation to participate in therapy and rehabilitation if they suffer from poor internal stigmatization [19]. Additionally, they may feel pressured to reject assistance and support from others due to internal stigma, which makes them believe they are a burden on their loved ones and the community.

Community culture significantly impacts individuals’ internal stigmas, which are self-negative perceptions caused by specific conditions, and these stigmas can impact their quality of life. Internal stigmatization is common among people who experience social humiliation as a result of their traits or illnesses. It can reduce their self-esteem and quality of life, making it more difficult for them to get help and ultimately reducing their quality of life [20].

### 1.2. Objective

In order to evaluate the concept and reliability of the SSS, a psychometric test would be conducted with specific patients in Indonesia with stroke. The purpose of this research was to investigate the reliability and validity of the Indonesian version of SSS (SSS-I) among stroke patients in Indonesia.

## 2. Materials and Methods

### 2.1. Study Design

There were two parts to the study: the translation process, using the Brislin method, and the psychometric testing of the SSS in Indonesia through a cross-sectional approach to ensure its reliability and construct validity.

### 2.2. Cultural Adaptation and Translation of the Questionnaire

The original developer was informed and provided full consent prior to initiating the translation of the SSS. Following the approval, the SSS was translated from English into Indonesian using Brislin’s back-translation technique [21]. Initially, the instrument underwent forward-translation from English into Indonesian, carried out by a sworn translator and a professor of nursing specializing in cultural studies and translation. Subsequently, five registered nurses holding doctoral degrees in adult nursing care, alongside a certified translator, reviewed the translation to identify grammatical errors and phrases that were unclear or difficult to comprehend. In the next phase, two translators—a nursing neurology associate professor and a sworn translator—performed back-translation from Indonesian to English. Additionally, two bilingual translators independently conducted another back-translation without having access to the original English version. Finally, both the original and revised versions underwent an evaluation for conceptual equivalence. An expert panel involved throughout the translation confirmed that both versions were conceptually identical and free from grammatical or semantic errors [21].

In order to establish the semantic equivalency between the original English and final Indonesian versions of the SSS instrument, five additional experts—who were not involved in the translation process—evaluated the translation equivalency between each question. Using a Likert scale that goes from 1 (very similar) to 4 (moderately similar) to 7 (not at all similar), we were able to evaluate the degrees of linguistic and interpretive similarity [22]. Words, phrases, and sentences that are formally similar are considered to be comparable in language. The similarity in interpretability can be defined as the extent to which two versions with different terminology elicit the same response. A formal review of the translation is required when the average score is more than 3 (with 7 representing the least amount of agreement and 1 the most), and reviewers did not consider there to be a serious interpretation problem when the score was less than 3 [22].

After the Indonesian translation was obtained, a pilot study was conducted to assess the questionnaire on 30 stroke patients with similar characteristics to the assigned group. A dichotomous scale with clear and unclear options was used to rate the questions, and every participant had to use it. There were no questions in the questionnaire that were difficult to understand. All things considered, it seems reasonable to conclude that the items were valid.

### 2.3. Psychometric Property Testing

#### 2.3.1. Participants and Study Settings

Using a simple random sampling method, participants were recruited from five different hospitals in Bali, Indonesia, between August and October 2024. The inclusion criteria were (1) adults with a history of surviving from stroke, (2) ≥18 years, (3) fluent in spoken and written Bahasa Indonesian, (4) free of aphasia and other mental health diagnoses.

For cross-cultural research, it is recommended to have at least seven participants per item, taking into account the sample size [23]. The validity and reliability test for SSS, which consists of 16 items, requires a total of 112 participants.

#### 2.3.2. Instrument

The SSS was developed by Zhu et al. in 2019 to assess stigma among stroke survivors. The scale consists of 16 questions grouped into four key dimensions: physical impairment (4 items), social isolation (3 items), discrimination experience (4 items), and internalized stigma (5 items). Respondents rate their stigma-related experiences over the past seven days using a Likert scale from 1 to 5. Scores on the SSS can range from 16 to 80, with higher scores indicating greater levels of perceived stigma [14]. The scale has demonstrated strong validity and reliability, with a Cronbach’s alpha of 0.92 and Pearson correlation coefficients between 0.73 and 0.90 [14].

#### 2.3.3. Study Process

Some of the ways in which the Indonesian version of the SSS was evaluated for its translation and psychometric analysis were as follows. First, the translation was carried out according to Brislin’s back-translation paradigm for cross-cultural research. Second, a pilot study with 30 adults with stroke was carried out to make the final draft more readable and accurate. Third, we looked at the final version (content validity index, or CVI). Finally, we used psychometric analysis to determine the concept validity, convergent validity, and internal consistency, as shown in Table 1.

### 2.4. Data Analysis

After finalizing the Indonesian version of the SSS, content validity was evaluated to support the validity of the measurement tool. Five experts assessed the content validity index (CVI), calculating both the item-level CVI (I-CVI) and the scale-level CVI (S-CVI). Using a 4-point Likert scale (1 = not relevant, 2 = somewhat relevant, 3 = quite relevant, 4 = highly relevant), the I-CVI was computed as the proportion of experts rating each item as “highly relevant.” Items with an I-CVI > 0.79 were considered relevant, those between 0.70 and 0.79 required revision, and items below 0.70 were removed. Similarly, the researchers computed the S-CVI/UA by counting all items that achieved an I-CVI score of 1 and then dividing this total by the number of items overall. Meanwhile, the S-CVI/Ave emerged from averaging the individual I-CVI scores. Content validity is considered excellent when the S-CVI/UA reaches or exceeds 0.8 and the S-CVI/Ave attains a minimum threshold of 0.9 [24].

LISREL 8.72, Scientific Software International, Inc (Chapel Hill, NC, USA) was used to conduct confirmatory factor analysis (CFA) to assess the construct validity. CFA is typically used to test theoretical models explaining the variance among a set of items. Given that the SSS was initially based on the original instruments, CFA was essential to confirm the appropriateness of the original model structure. Acceptable CFA parameters included a non-significant chi-square (*p* > 0.05), chi-square/df < 3.00, CFI > 0.90, RMSEA < 0.07, SRMR < 0.08, GFI > 0.80, and AGFI > 0.80. Factor loadings were considered adequate if greater than 0.3 [25].

Convergent validity was assessed using the average variance extracted (AVE), which measures how much variance is captured by the latent construct relative to total variance. An AVE > 0.5 indicates a latent construct as it explains at least half the variance [26,27].

Reliability testing primarily utilized Cronbach’s alpha and composite reliability to assess internal consistency. Cronbach’s alpha values ≥0.7 indicate good internal consistency, while composite reliability values ≥0.7 confirm that the indicator variables adequately represent the latent construct [28]. Corrected item-total correlations, computed to examine each item’s association with the total scale score, used a threshold of >0.2 to retain important items relevant to a heterogeneous stroke population [29,30].

Data were analyzed with SPSS version 25, IBM Corp. (Armonk, NY, USA), checking for missing values, outliers, and normality. Categorical variables were reported as numbers and percentages, and continuous variables as means and standard deviations. The dataset had no missing values or outliers.

## 3. Results

### 3.1. Translation

The translation procedure went through four phases. Initially, two Indonesian bilingual specialists translated the SSS from English into Indonesian (a certified translator and professor in Nursing Science). Second, it was compared and discussed until a consensus was reached. Third, two experts who were proficient in both Indonesian and English independently back-translated the Indonesian version into English with cultural adjustments. An expert group looked over and contrasted the translated and back-translated versions. In order to assess each item in terms of its explicit construct definition and investigate its conceptual, experiential, idiomatic, and semantic equivalency, expert validation was carried out. A panel discussion resulted in an agreement being achieved to determine the final SSS-I [21]. Based on the translation process results, it was indicated that the interpretations of the original and Indonesian versions of the scale were equivalent (Figure S1).

The linguistic comparability of the SSS-I was assessed on Sperber’s 1–7 scale, on which scores of 1–2 indicate acceptable comparability [22]. The final SSS-I obtained was a 1.48 linguistic comparability score and 1.42 interpretative similarity. Similarly, the interpretative similarity was within the acceptable range of 1 to 2, indicating no significant issues. Since the scores satisfy predetermined acceptance, additional revisions or further assessment were unnecessary.

Five specialists, including two nurses specializing in neurology, one psychologist, and two psychiatric nurses, conducted the content validity evaluation. The I-CVI scores for the SSS-I were highly relevant, ranging from 0.94 to 1, and the S-CVI score was around 0.95, signifying excellent content validity for the SSS-I.

### 3.2. Psychometric Properties of SSS-I

#### Characteristics of Participants

The average age of the study participants was 61.36 ± 10.06 years. The duration of surviving from stroke averaged 2.35 ± 1.91 years. The majority were male (n = 62, 55.4%) with a senior high school education (36.6%), followed by those with no education (19.6%). Most of the participants had problems with the left arm and leg (53%) and lived with their children (31.3%). Most of the study participants were taking an antihypertensive medication (n = 79, 70.5%) to control their blood pressure.

### 3.3. Construct Validity

The unidimensional model did not fit well with the empirical data (see Table 1). Meanwhile, the original concept of the SSS showed acceptable model fit, as shown in Table 1: χ^2^ (0.00), χ^2^/df (1.724), CFI (0.97), RMSEA (0.077), SRMR (0.064), GFI (0.85), and AGFI (0.79). Additionally, by creating correlation trajectories between item errors, a modified model was created [31]. The modified model showed that there was no significant change compared to the initial model. The initial model was confirmed as representing an acceptable fit for the SSS-I. It is noted that GFI (0.85) fell marginally below the conventional threshold of 0.90, AGFI (0.79) fell just below the 0.80 threshold, and RMSEA (0.077) approached the upper boundary of acceptable fit; these indices should therefore be interpreted with appropriate caution.

**Figure 1:**
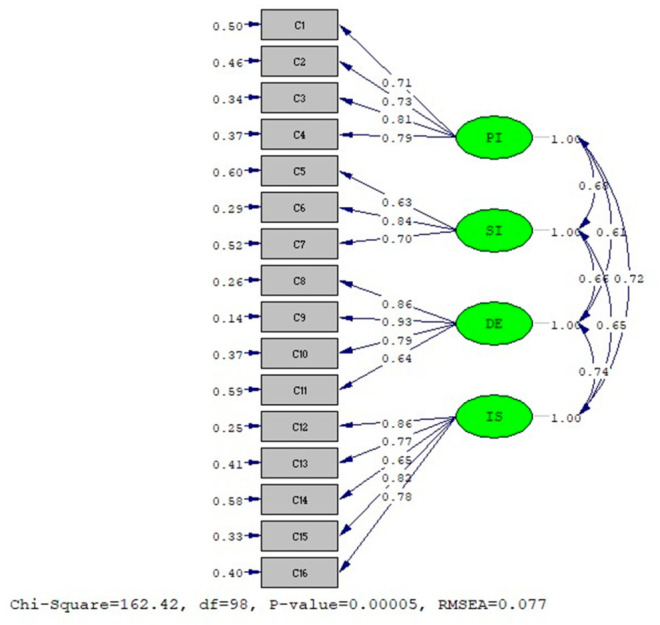
Initial Measurements Model.

According to the confirmatory factor analysis (CFA), the Indonesian version of the SSS-I demonstrated statistically significant results across all four dimensions. The standardized factor loadings within each dimension ranged from 0.63 to 0.93. Specifically, factor loadings varied between 0.71 and 0.79 for physical impairment, 0.63 and 0.84 for social isolation, 0.64 and 0.93 for discrimination experience, and 0.65 and 0.86 for internalized stigma. Figure 1 illustrates the initial measurement model.

### 3.4. Convergent Validity

The AVE values ranged from 0.507 to 0.666, demonstrating acceptable convergent validity. This indicates that the latent constructs accounted for more than 50% of the variance in their indicators.

### 3.5. Reliability

Internal consistency was assessed using Cronbach’s alpha, with an overall coefficient of 0.917 for the SSS-I. The Cronbach alpha scores for individual dimensions were 0.778 (physical impairment), 0.673 (social isolation), 0.862 (discrimination experience), and 0.866 (internalized stigma). The corrected item-total correlation coefficients for each dimension ranged from 0.419 to 0.838 (Table 2). Additionally, composite reliability, also known as construct reliability, ranged from 0.752 to 0.887 for each latent variable, indicating good to excellent reliability.

## 4. Discussion

To the best of our knowledge, this is the first study carried out by a team of academics and professionals from diverse disciplines to evaluate the psychometric properties of the Indonesian version of the SSS among individuals who have survived strokes in Indonesia. Three processes were followed comprising translation, validity, and reliability. Regarding the translation procedure, some words were regarded as having multiple meanings in the back-translation process. The translation process of the SSS into Indonesian SSS-I adhered to established best practices for the cross-cultural adaptation of measurement tools. The four-phase translation process was guided by Brislin’s model, ensuring conceptual, idiomatic, semantic, and experiential equivalence [21]. The linguistic comparability score aligns with Sperber’s criteria, indicating that the SSS-I maintains equivalency with the original instrument [22]. Furthermore, content validity was confirmed by expert evaluation, surpassing the commonly accepted threshold of 0.80 [32].

Construct validity was assessed using CFA, a widely used method for evaluating the structural integrity of psychometric instruments [33]. The original conceptual model of the SSS exhibited an acceptable fit, with key indices—χ^2^/df (1.724), CFI (0.97), RMSEA (0.077), SRMR (0.064), GFI (0.85), and AGFI (0.79). While CFI and χ^2^/df met commonly recommended thresholds, GFI (0.85) fell below the conventional cut-off of 0.90 and AGFI (0.79) fell marginally below the 0.80 threshold, RMSEA (0.077) approached the upper boundary of acceptable fit. These values suggest acceptable rather than strong model fit and should be interpreted accordingly [34]. Although model modifications were attempted by correlating error terms [31], the initial model retained its goodness-of-fit, demonstrating robust structural validity.

Convergent validity was assessed using the AVE, with values ranging from 0.507 to 0.666. These results exceed the 0.50 threshold suggested by Fornell and Larcker, confirming that the latent constructs account for more than half of the variance in their indicators [27]. Composite reliability (CR) scores for the four subscales—physical impairment (0.838), social isolation (0.752), discrimination experience (0.887), and internalized stigma (0.886)—were well above the acceptable limit of 0.70 [25], reinforcing the construct validity of SSS-I.

Internal consistency was evaluated using Cronbach’s alpha, which yielded an overall reliability score of 0.917, surpassing the commonly accepted threshold of 0.70 for psychological scales [35]. The subscale reliability values—0.778 for physical impairment, 0.673 for social isolation, 0.862 for discrimination experience, and 0.866 for internalized stigma—demonstrate acceptable to good internal consistency. The social isolation subscale yielded a Cronbach’s alpha of 0.673, which falls marginally below the conventional threshold of 0.70; this may reflect the limited number of items (three) in this subscale and warrants cautious interpretation. The corrected item-total correlation values (0.419–0.838) further support the homogeneity of the scale items. Composite reliability (CR) scores between 0.752 and 0.887 confirm that the SSS-I possesses both high reliability and construct validity, making it a robust tool for assessing stigma among stroke survivors.

### Limitations

The current research has several important limitations. First, recruitment was restricted to hospital-registered stroke survivors, which may limit generalizability to those managed in primary care or community settings. Second, individuals with aphasia were excluded, despite aphasia being among the most common and disabling post-stroke consequences; this exclusion may substantially underestimate stigma burden in the broader stroke population. Third, the analytic sample (N = 112) is modest for a four-factor CFA, which may affect the stability and generalizability of the factor structure. Fourth, the intraclass correlation coefficient (ICC) for test–retest reliability was not assessed, limiting conclusions about temporal stability. Fifth, discriminant validity was not evaluated, and further studies examining the distinctiveness of the four subscales are needed. Sixth, the social isolation subscale Cronbach’s alpha (0.673) fell below the conventional 0.70 threshold. Finally, all participants were recruited from hospitals in Bali; multi-site studies across diverse Indonesian regions are needed to establish broader psychometric generalizability.

## 5. Conclusions

The findings of this study provide preliminary evidence supporting the validity and reliability of the SSS-I in Indonesia. The translation process adhered to established guidelines for linguistic and cultural adaptation, while the CFA results validated the structural integrity of the scale. The high content validity and strong internal consistency further reinforce its reliability as a measurement tool. Given these results, the SSS-I is a valuable instrument for assessing stroke-related stigma in Indonesia, and the SSS-I can be used for both research and clinical applications.

### 5.1. Implications for Practice

The validated Indonesian version of the SSS-I provides a reliable tool for assessing stigma among Indonesian stroke survivors, allowing nurses to identify psychological and social challenges that may impact recovery. Given that stroke-related stigma can contribute to social isolation, discrimination, and internalized negative self-perceptions, nurses play a crucial role in integrating stigma assessment into routine patient care. By recognizing stigma-related distress, nurses can implement targeted interventions, such as psychoeducation, peer support programs, and family counseling, to enhance patient well-being. Additionally, the findings support the need for culturally sensitive nursing interventions that address the specific experiences of Indonesian stroke survivors, promoting a holistic approach to rehabilitation.

Health workers should also advocate for multidisciplinary collaborations involving psychologists, social workers, and rehabilitation specialists to address the multifaceted impact of stigma. Training programs for nurses should incorporate stigma reduction strategies, emphasizing the importance of communication, empathy, and empowerment in patient interactions. Moreover, community-based nursing initiatives can help reduce stigma at a societal level by increasing awareness about stroke recovery and combating misconceptions.

### 5.2. Key Points for Policy, Practice and/or Research

1. Holistic rehabilitation should not only address physical recovery, but also psychological and social challenges caused by stigma.
2. Routine stigma assessment using tools like the Stroke Stigma Scale (SSS-I) can help identify hidden barriers to recovery.
3. Psychoeducation and counseling for patients and families can reduce misconceptions and foster better social support.
4. Peer support programs may enhance self-esteem and reduce feelings of isolation.
5. Multidisciplinary collaboration (nurses, psychologists, social workers, rehabilitation specialists) is essential to address stigma’s multifaceted effects.
6. Nurse training programs should integrate stigma reduction strategies, emphasizing empathy, communication, and empowerment.
7. Community-based interventions can raise awareness and reduce public stigma toward stroke survivors.
8. Policy integration into national stroke management should address stigma-related barriers to care and social reintegration.

## Figures and Tables

**Table 1:** SSS-I goodness of fit statistics (N = 112).

Relative Fit Index	Unidimensional Model	Initial Model	Modified Model	Acceptable Goodness of Fit Statistics
Chi-square test	<0.001	<0.001	0.062	*p* > 0.05
Chi-square test/degree of freedom	3.378	1.724	1.268	<3.00
CFI	0.91	0.97	0.99	>0.90
RMSEA	0.016	0.077	0.046	<0.08
SRMR	0.087	0.064	0.057	<0.07
GFI	0.69	0.85	0.88	>0.90
AGFI	0.59	0.79	0.83	>0.80

**Table 2:** Summary of the construct reliability, average variance extracted, and internal consistency.

Variable	Item	Construct Reliability of Latent Variables	AVE	Cronbach’s α	Item to Total Correlation
PI	4	0.838	0.564	0.778	0.504–0.676
SI	3	0.752	0.507	0.673	0.419–0.590
DE	4	0.887	0.666	0.862	0.602–0.838
IS	5	0.886	0.611	0.866	0.566–0.804
Total	16			0.917	0.462–0.753

PI = Physical Impairment, SI = Social Isolation, DE = Discrimination Experience, IS = Internalized Stigma.

## Data Availability

The datasets generated during and/or analyzed during the current study are not publicly available due to patient requirements, but are available from the corresponding author upon reasonable request.

## Supplementary Materials

The following supporting information can be downloaded at https://www.mdpi.com/article/10.3390/ijerph23070918/s1. Figure S1: Participant screening, enrollment, and analysis flowchart. Of 190 stroke survivors initially assessed for eligibility, 78 were excluded (42 did not meet inclusion criteria, including aphasia (n = 24), a low score on the Mental State Examination (n = 11), and other criteria not met (n = 7); 21 declined to participate; and 15 returned incomplete questionnaires). The remaining 112 participants were enrolled and underwent content validity review by an expert panel (n = 5), followed by pilot testing in a subsample (n = 30) to assess item comprehensibility. All 112 participants completed the main data collection and were included in the final psychometric analysis, comprising confirmatory factor analysis, reliability assessment, and convergent validity testing.

## References

[1] Feigin V.L., Brainin M., Norrving B., Martins S.O., Pandian J., Lindsay P., Grupper M.F., Rautalin I. (2025). World Stroke Organization: Global Stroke Fact Sheet 2025. Int. J. Stroke.

[2] Goffman E. (1963). Stigma: Notes on the Management of Spoiled Identity.

[3] Andersen M.M., Varga S., Folker A.P. (2022). On the definition of stigma. J. Eval. Clin. Pract..

[4] Brigiano M., Calabrese L., Chirico I., Trolese S., Quartarone M., Forte L., Annini A., Murri M.B., Chattat R. (2025). Within My Walls, I Escape Being Underestimated: A Systematic Review and Thematic Synthesis of Stigma and Help-Seeking in Dementia. Behav. Sci..

[5] Kariasa I.M., Aungsuroch Y., Nurachmah E., Nova P.A., Dewi N.L.P.T., Juanamasta I.G., Polsook R. (2024). Factors Influencing Stroke Internal Stigma Among Stroke Survivors. SAGE Open Nurs..

[6] Yuniarti I.I. (2022). Factors Affecting Self Management in Stroke Patients. Master’s Thesis.

[7] Kitamura S., Miyamoto R., Watanabe S., Yoshida T., Ishii Y. (2024). Development of the Japanese version of the stroke stigma scale: A validity and reliability assessment. Top. Stroke Rehabil..

[8] Cheong M.J., Kang Y., Kang H.W. (2021). Psychosocial Factors Related to Stroke Patients’ Rehabilitation Motivation: A Scoping Review and Meta-Analysis Focused on South Korea. Healthcare.

[9] Dewi N.L.P.T., Kariasa I.M., Yundari A.I.D.H., Pendet N.M.D.P., Juanamasta I.G. (2024). Factors influencing self-management for preventing recurrent stroke attacks among patients at the stroke foundation clinic in Bali, Indonesia, 2023. Nurs. Midwifery Stud..

[10] Elendu C., Amaechi D.C.M., Elendu T.C.B., Ibhiedu J.O.M., Egbunu E.O.M., Ndam A.R.M., Ogala F.M., Ologunde T.M., Peterson J.C.M., Boluwatife A.I.M. (2023). Stroke and cognitive impairment: Understanding the connection and managing symptoms. Ann. Med. Surg..

[11] Murphy S.J., Werring D.J. (2020). Stroke: Causes and clinical features. Medicine.

[12] Werring D., Adams M., Benjamin L., Brown M., Chandratheva A., Cowley P., Grieve J., Humphries F., Jäger H.R., Losseff N., Howard R., Kullmann D., Werring D., Zandi M. (2024). Stroke and Cerebrovascular Diseases. Neurology.

[13] Kim J.S. (2016). Post-stroke Mood and Emotional Disturbances: Pharmacological Therapy Based on Mechanisms. J. Stroke.

[14] Zhu M., Zhou H., Zhang W., Deng Y., Wang X., Bai X., Li M., Hu R., Hou J., Liu Y. (2019). The Stroke Stigma Scale: A reliable and valid stigma measure in patients with stroke. Clin. Rehabil..

[15] Kalavina R., Chisati E., Mlenzana N., Wazakili M. (2019). The challenges and experiences of stroke patients and their spouses in Blantyre, Malawi. Malawi Med. J..

[16] Göksu E.Ö., Katı Ş.D. (2021). Internal Stigmatization and Mental Health in Patients with Stroke. Turk. J. Neurol..

[17] Zhu M., Zhou H., Zhang W., Deng Y., Wang X., Zhang X., Yang L., Li M., Bai X., Lin Z. (2019). Stigma experienced by Chinese patients with stroke during inpatient rehabilitation and its correlated factors: A cross-sectional study. Top. Stroke Rehabil..

[18] Deng C., Lu Q., Yang L., Wu R., Liu Y., Li L., Chen S., Wei S., Wang Y., Huang Y. (2019). Factors associated with stigma in community-dwelling stroke survivors in China: A cross-sectional study. J. Neurol. Sci..

[19] Fan W., Ma K.K., Yang C.X., Guo Y.L. (2023). The mediating effect of stigma between self-perceived burden and loneliness in stroke patients. Front. Psychiatry.

[20] Harris C. (2023). Breaking the Cycle of Stigma: The Role of Majority Group Stigmatization in Contributing to Internalized Stigma Among Racial Minorities. Bachelor’s Thesis.

[21] Brislin R.W. (1970). Back-translation for cross-cultural research. J. Cross-Cult. Psychol..

[22] Sperber A.D. (2004). Translation and validation of study instruments for cross-cultural research. Gastroenterology.

[23] Prinsen C.A.C., Mokkink L.B., Bouter L.M., Alonso J., Patrick D.L., de Vet H.C.W., Terwee C.B. (2018). COSMIN guideline for systematic reviews of patient-reported outcome measures. Qual. Life Res..

[24] Kimberlin C.L., Winterstein A.G. (2008). Validity and reliability of measurement instruments used in research. Am. J. Health-Syst. Pharm..

[25] Hair J.F., Black W.C., Babin B.J., Anderson R.E. (2010). Multivariate Data Analysis.

[26] Carlson K.D., Herdman A.O. (2012). Understanding the Impact of Convergent Validity on Research Results. Organ. Res. Methods.

[27] Fornell C., Larcker D.F. (1981). Evaluating Structural Equation Models with Unobservable Variables and Measurement Error. J. Mark. Res..

[28] Heale R., Twycross A. (2015). Validity and reliability in quantitative studies. Evid. Based Nurs..

[29] Boonyaratana Y., Hansson E.E., Granbom M., Schmidt S.M. (2021). The Psychometric Properties of the Meaning of Home and Housing-Related Control Beliefs Scales among 67–70 Year-Olds in Sweden. Int. J. Environ. Res. Public Health.

[30] Ferketich S. (1991). *Focus on psychometrics*. Aspects of item analysis. Res. Nurs. Health.

[31] Saurina C., Coenders G. (2002). Predicting Overall Service Quality: A Structural Equation Modelling Approach. Metodološki Zvezki.

[32] Polit D.F., Beck C.T. (2018). Essentials of Nursing Research: Appraising Evidence for Nursing Practice.

[33] Kline R.B. (2016). Principles and Practice of Structural Equation Modeling.

[34] Hu L.T., Bentler P.M. (1999). Cutoff criteria for fit indexes in covariance structure analysis: Conventional criteria versus new alternatives. Struct. Equ. Model..

[35] Nunnally J.C., Bernstein I. (1978). Psychometric Theory.

