# Supplementary material for: Evaluating the Psychometric Properties of the Indonesian Version of the Stroke Stigma Scale Among Indonesian Stroke Survivors
Source: Int J Environ Res Public Health. 2026 Jul 17;23(7):918. doi: 10.3390/ijerph23070918 (PMC13410054; doi:10.3390/ijerph23070918)
Supplement: Supplementary file 1 [file ijerph-23-00918-s001.zip › ijerph-4218566-supplementary.pdf]

Supplementary Materials

Evaluating the Psychometric Properties of the Indonesian Version of the Stroke Stigma Scale among Indonesian Stroke Survivors

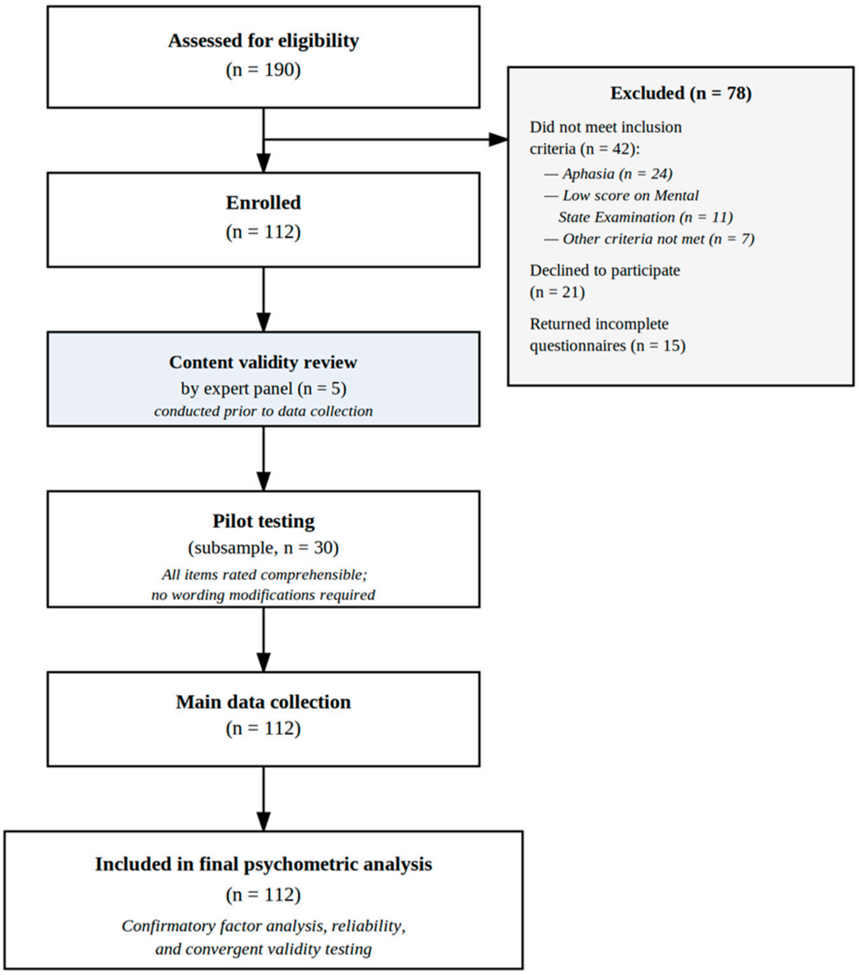

Participant screening, enrollment, and analysis flowchart (CONSORT-style).

CFA = confirmatory factor analysis.

Figure S1. Participant screening, enrollment, and analysis flowchart. Of 190 stroke survivors initially assessed for eligibility, 78 were excluded (42 did not meet inclusion criteria, including aphasia (n = 24), a low score on the Mental State Examination (n = 11), and other criteria not met (n = 7); 21 declined to participate; and 15 returned incomplete questionnaires). The remaining 112 participants were enrolled and underwent content validity review by an expert panel (n = 5), followed by pilot testing in a subsample (n = 30) to assess item comprehensibility. All 112 participants completed the main data collection and were included in the final psychometric analysis, comprising confirmatory factor analysis, reliability assessment, and convergent validity testing.
